# An improved draft of the pigeonpea (*Cajanus cajan* (L.) Millsp.) genome

**DOI:** 10.1016/j.dib.2017.11.066

**Published:** 2017-11-22

**Authors:** Ajay Kumar Mahato, Ajay Kumar Sharma, Tilak Raj Sharma, Nagendra Kumar Singh

**Affiliations:** a ICAR-National Research Centre on Plant Biotechnology (NRCPB), IARI, Pusa Campus, New Delhi 110012, India; b Meerut Institute of Engineering and Technology, A.P.J. Abdul Kalam Technical University (APJAKTU), Meerut, Uttar Pradesh 226031, India

## Abstract

The first draft of the pigeonpea (*Cajanus cajan* (L.) Millsp. cv. Asha) genome with 511 Mbp of assembled sequence information has low genome coverage of about sixty percent. Here we present an improved version of this genome with 648.2 Mbp of assembled sequence of this popular pigeonpea variety, which is liked by the millers and has resistance to fusarium wilt and sterility mosaic diseases. With the addition of 137 Mb of assembled sequence information the present version has the highest available genome coverage of pigeonpea till date. We predicted 56,888 protein-coding genes of which 54,286 (96.7%) were functionally annotated. In the improved genome assembly we identified 158,432 SSR loci, designed flanking primers for 85,296 of these and validated them *in-silico* by e-PCR. The raw data used for the improvement of genome assembly are available in the SRA database of NCBI with accession numbers SRR5922904, SRR5922905, SRR5922906, SRR5922907. The genome sequence update has been deposited at DDBJ/EMBL/GenBank under the accession AFSP00000000, and the version described in this paper is the second version (AFSP02000000).

**Specifications Table**

**Table:** 

Subject area	Biology
More specific subject area	Legume, Genomics
Type of data	Sequence reads, Genome assembly, SSR markers
How data was acquired	Shotgun whole-genome Illumina HiSeq sequencing platform, *de novo* assembly and computational merging of different assemblies, computational SSR mining
Data format	Raw, Analyzed
Experimental factors	Adapters along with poor quality bases removed from the genomic sequence reads
Experimental features	Illumina sequence data which after de novo assembly and merging with first draft of pigeonpea genome resulted an improved draft version of pigeonpea genome, SSR identification and primer designing
Data source location	ICAR-National Research Centre on Plant Biotechnology, Indian Agricultural Research Institute, New Delhi, India
Data accessibility	The improved draft genome assembly of pigeonpea is available at DDBJ/ENA/GenBank under the accession number AFSP00000000. The version described in this paper is second version AFSP02000000 (https://www.ncbi.nlm.nih.gov/nuccore/AFSP02000000)
	The raw Illumina sequence reads are available at NCBI under the SRA database accession number SRR5922904 (https://www.ncbi.nlm.nih.gov/sra/?term=SRR5922904), SRR5922905 (https://www.ncbi.nlm.nih.gov/sra/?term=SRR5922905), SRR5922906(https://www.ncbi.nlm.nih.gov/sra/?term=SRR5922906), SRR5922907 (https://www.ncbi.nlm.nih.gov/sra/?term=SRR5922907). SSR flanking primer sequence data available with this article

**Value of the data**

• Provides an updated and much improved draft genome assembly of pigeonpea.
• Provides genome wide SSR marker information that can be used to target highly variable regions across the pigeonpea varieties and other closely related taxa for breeding applications.

## 1 Data

**Fig. 1:**
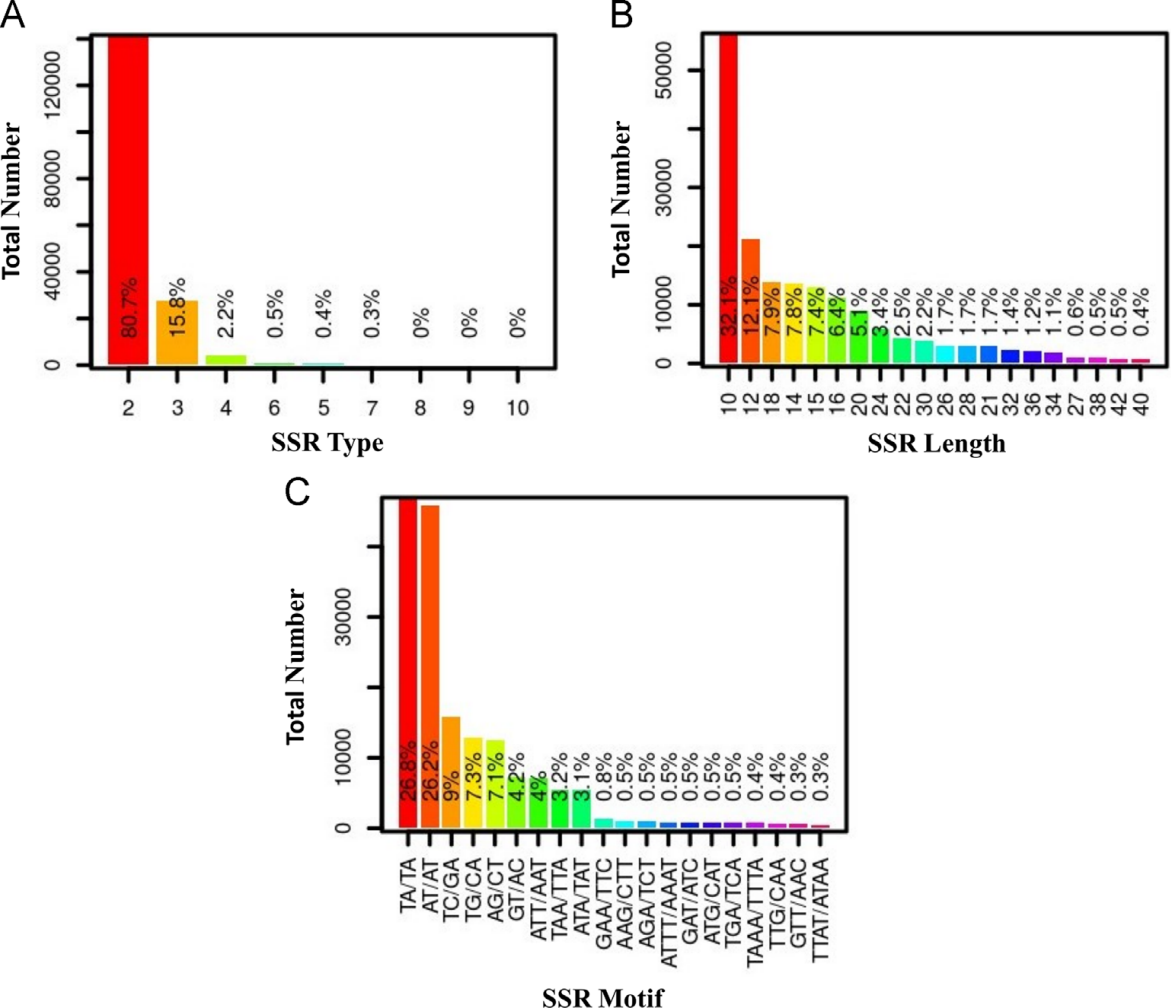
**SSR identification result; (**Fig. 1**-A)** Frequency of different types of SSR identified; (2=Di, 3=Tri, 4=Tetra, 5=Penta, 6=Hexa, 7=Hepta, 8=Octa, 9=Nona and 10=Deca nucleotide SSR types) (Fig. 1**-B)** Distribution of identified SSR over different SSR length (bp); (Fig. 1**-C)** Abundance distribution of top 20 SSRs motif groups in total predicted SSRs.

We present an improved draft genome assembly of pigeonpea having estimated total genome size of 858 Mb [1]. Pigeonpea is the fourth most important food legume, and owing to high protein, mineral and vitamin contents it is playing significant role in the eradication of protein-calorie malnutrition in Asia and Africa [2]. The Illumina HiSeq sequence reads generated in this study have been deposited in the NCBI-SRA database (SRR5922904, SRR5922905, SRR5922906, SRR5922907) and improved draft genome of pigeonpea is deposited in the NCBI-WGS database (AFSP02000000). Data presented in the text includes tables and figures providing information on the different library types of Illumina sequence data (Table 1), statistics of improved draft genome assembly (Table 2) as well as identified genome wide SSRs (Fig. 1) along with their flanking primer sequence information (Supplementary Table 1).

**Table 1:** Summary of next generation sequence (NGS) data generated to improve the draft genome assembly of pigeonpea.

**Data Type**	**Total Raw Reads**	**Total Raw bases**	**High Quality Reads**	**Total HQ bases**
**Illumina Single End**	659,378,791	65,937,879,100	575,811,750	57,624,312,918
**Illumina Paired End**	220,817,189	44,163,437,800	201,296,367	39,355,530,995
**Illumina Mate Pair 3Kb**	176,384,139	35,276,827,800	158,950,418	30,892,669,789
**Illumina Mate Pair 5Kb**	199,824,068	39,964,813,600	179,942,128	35,097,467,239
**TOTAL**	1,256,404,187	185,342,958,300	1,116,000,663	162,969,980,941

**Table 2:** Summary statistics of improved draft genome assembly of pigeonpea.

**S. No.**	**Assembly Details**	**First draft assembly (Roche-454)**	**Present assembly (Illumina)**	**Improved merged Assembly**
1	Total Number of Contigs	191,705	364,837	360,028
2	Total Number of bases	510,809,477	550,390,714	648,281,402
3	Largest contig size	45,193	118,005	183,327
4	Mean contig size	2661	1509	1801
5	N50	4522	5030	5341
6	Number of contigs > 1 Kbp	127,907 (66.7%)	96,359 (26.8%)	121,393 (33.7%)
7	Number of contigs > 10 Kbp	6953 (3.6%)	10,142 (2.8%)	11,833 (3.3%)
9	contig % A	32.88	33.09	33.21
10	contig % C	16.72	16.92	16.73
11	contig % G	16.97	16.91	16.76
12	contig % T	33.44	33.08	33.29

## 2 Experimental design, material and methods

### 2.1 Plant material, DNA isolation and genome sequencing

High quality DNA was isolated from the leaves of pigeonpea variety ‘Asha’ using CTAB method [3]. DNA was fragmented with a median fragment size of 350 bp, 550 bp, 3 Kb and 5 Kb and used for whole genome shotgun, paired-end and mate-pair sequencing using Illumina HiSeq-2000 sequencing platform (Illumina, San Diego, CA).

### 2.2 Genome sequencing, de-novo assembly and gene annotation

The Illumina sequence reads were quality-checked using FASTQC (http://www.bioinformatics.babraham.ac.uk/projects/fastqc/) and adapter sequences along with poor-quality bases were removed using Trimmomatic v 0.36 [4] (Table 1). The high-quality Illumina reads were *de novo* assembled using software CLC Genomics Workbench version 7.1 (CLC Bio, Aarhus, Denmark, http://www.clcbio.com/). The improved draft version of assembly (Table 2) was generated using software GAM-NGS [5] by merging the first draft 454- GS-FLX sequence based assembly [6] with the new Illumina based draft assembly.

The improved merged draft assembly consists of 360,028 contigs with total size of 648.2 Mb and covers 75.6% of the genome, which is 15% higher than the published first draft genome of pigeonpea [6], and 11% higher than another published draft of pigeonpea [7]. To predict the protein coding genes the improved draft assembly was first repeat-masked using RepeatModler and RepeatMasker software [8], followed by *ab-initio* gene prediction using the FGENESH module of the Molquest v. 4.5 software package (http://www.softberry.com). The predicted genes were annotated using BLASTX (*E*<10^-6^) [9] search against the NCBI non-redundant (nr) protein database using Blast2GO software [10].

### 2.3 Identification of genome wide SSR and designing of PCR primers

The improved draft version was screened for the presence of simple sequence repeat (SSR) loci using MISA software (http://pgrc.ipk-gatersleben.de/misa/), the output is tabulated and graphically represented in Fig. 1. The SSR flanking primer sequences were designed with the help of Primer3 software [11] and efficiency of primer specificity was checked using software e-PCR [12]. The complete details of the SSR primers are available in Supplementary Table 1.

## Appendix A Supplementary material

**Supplementary Table 1**. Complete details of SSRs primers. Supplementary material
