# Supplementary material for: An improved draft of the pigeonpea (Cajanus cajan (L.) Millsp.) genome
Source: Data Brief. 2017 Nov 22;16:376–80. doi: 10.1016/j.dib.2017.11.066 (PMC5723258; doi:10.1016/j.dib.2017.11.066)
Supplement: Supplementary file 1 — Supplementary material [file mmc1.pdf]

Conflict of Interest Form

We confirm that the manuscript has been read and approved by all named authors and that there are no other persons who satisfied the criteria for authorship but are not listed. We further confirm that the order of authors listed in the manuscript has been approved by all of us.

Signed by all authors as follows:

S. No.	Author Name	E-mail ID	Date	Signature
1	Ajay Kumar Mahato	ajaybioinfo@gmail.com	11/10/2017	Ajay
2	Ajay Kumar Sharma	aks.ajayksharma@gmail.com	12/10/2017	[illegible]
3	Tilak Raj Sharma	trsharma1965@gmail.com	13/10/17	[illegible]
4	Nagendra Kumar Singh	nksingh4@gmail.com	11/10/17	[illegible]
